# The efficacy of Chinese herbal medicine as an adjunctive therapy for colorectal cancer: A protocol for systematic review of randomized controlled trials

**DOI:** 10.1097/MD.0000000000023216

**Published:** 2020-12-18

**Authors:** Wenyuan Li, Jing Guo, Qiaoling Wang, Jianyuan Tang, Fengming You

**Affiliations:** a Hospital of Chengdu University of Traditional Chinese Medicine; b Evidence-Based Traditional Chinese Medicine Center of Sichuan Province, No.39 Shi-er-qiao Road, Chengdu, Sichuan Province, P.R. China.

**Keywords:** adjunctive therapy, Chinese herbal medicine, colorectal cancer, randomized controlled trials, systematic review

## Abstract

**Background:**

Colorectal cancer (CRC) is a public health problem and the world's leading cancer killer. It is a disease with high incidence and mortality. Although chemotherapy has achieved some success in the treatment of CRC, drug resistance and tumor metastasis caused by chemotherapy are still the main causes of death in patients with CRC. Notably, many side effects associated with chemotherapy, such as nausea, vomiting, and peripheral neurotoxicity, are major challenges in the treatment of patients with CRC. Chinese herbal medicine (CHM) has been widely used as an adjunctive therapy for CRC, but its efficacy and safety are still uncertain. The aim of this systematic review is to assess the efficacy and safety of CHM for the treatment of CRC.

**Methods:**

A comprehensive retrieval will be performed in the following electronic databases: PubMed, Cochrane Library, EMBASE, Web of Science, CNKI, SinoMed, VIP, and Wan Fang Data. The methodologic quality of randomized controlled trials will be assessed using the Cochrane risk assessment tool. Review Manager 5.3 software will be used for data synthesis and analysis. Funnel plot analysis and Egger test will be used to assess publication bias. The Grading of Recommendations Assessment, Development and Evaluation standard will be used to generate summary of finding table.

**Results:**

The results of this systematic review will be used to summarize and evaluate the evidence from randomized controlled clinical trials of CHM as adjuvant therapy for CRC.

**Conclusion:**

This review will provide a detailed summary of the evidence to assess the efficacy and safety of CHM for CRC.

**OSF Registration:**

DOI 10.17605/OSF.IO/X2SKJ

## 1 Introduction

Nowadays, colorectal cancer (CRC) is 1 of the most common malignant tumors of the digestive tract worldwide and remains a serious threat to human health. It is characterized by high incidence, high mortality, and unfavorable prognosis. Unfortunately, when diagnosed, a large percentage of patients have metastases or relapse after a few months, thus resulting in poor prognosis.^[1]^ It is reported that the 5-year survival rate for metastatic CRC remains disappointing at approximately 10%.^[2]^ Age, genetic and environmental factors play a major role in the development of CRC.^[3]^ CRC is a real concern. Although CRC is most likely to occur over the age of 65, epidemiological studies have shown that the average age of patients with CRC is continuing to decline and that the incidence of CRC is increasing among people under 50.^[4]^ In other words, more and more young people are suffering from this so-called geriatric disease. As Chinese people became richer and shift to meat-based diets and lifestyles, the incidence and mortality of CRC gradually increased year by year in China.^[5]^ Despite effective cancer screening measures, early interventions, better treatment options, the incidence and the mortality rate of CRC have declined.^[6,7]^ But according to the 2018 China Cancer Statistics Report, the incidence and mortality of CRC in China ranked third and fifth among all malignant tumors, respectively, with 376,000 new cases and 191,000 deaths.^[8]^

Chemotherapy is regarded as a standard treatment for CRC and plays a key role in improving prognosis. It is effective in inhibiting tumor growth, reducing tumor size and delaying disease progression.^[9]^ However, limitations of chemotherapy cannot be ignored, such as low selectivity, insufficient concentrations in tumor tissues, and systemic toxicity.^[10]^ Chemotherapy is also associated with various side effects, including nausea, vomiting, peripheral neuropathy, and diarrhea, which thereby restrict its clinical application, reduce the quality of life (QOL) of patients, and lead to patients’ intolerance to chemotherapy. Although various treatment strategies have made progress in managing chemotherapy side effects, these still hardly satisfy the requirements of patients undergoing chemotherapy.^[11–13]^ Thus, there is an urgent need to develop alternative therapy to manage these side effects.

Chinese herbal medicine (CHM) has a long history in China and is gaining popularity as a form of complementary and alternative medicine. CHM has developed over thousands of years with a unique system of theories, diagnostics, and treatments. Currently, CHM has been widely accepted and used in the adjuvant therapy of various cancers in China. CHM formulas also have been increasingly used as an adjuvant therapy to manage chemotherapy-induced side effects and improve the completion rate of chemotherapy.^[14–16]^ Current evidence from preclinical studies also suggests that CHM is beneficial in preventing or alleviating chemotherapy-induced side effects.^[17]^ Moreover, some researches have shown that CHM may plays a significant role in tumorigenesis, reduction of toxicity, alleviating clinical symptoms, prolonging survival time and decreasing the risk of CRC recurrence and metastasis.^[18]^ To our knowledge, few systematic reviews are evaluating the efficacy of CHM for CRC, it's still necessary to summarize the available evidence to support informed decision making.

In this systematic review, we aim to focus on the role of CHM in CRC. We hope to evaluate the efficacy of CHM combined with chemotherapy for CRC through randomized controlled trials on the evidence of overall survival (OS), survival rate, progression-free survival, objective response rate, side effects associated with chemotherapy, QOL and adverse effects.

## 2 Methods

### 2.1 Protocol registrations

This systematic review protocol has been registered prospectively in the OSF Registration (https://osf.io) and the registration number is DOI 10.17605/OSF.IO/X2SKJ. The procedure of this protocol will be reported strictly by the Preferred Reporting Items for Systematic Review and Meta-Analysis Protocols.^[19]^

### 2.2 Database and search strategy

The following online databases will be searched for all relevant studies from their inception up to November 2020: Cochrane Library, PubMed, EMBASE, Web of Science, China National Knowledge Infrastructure Database (CNKI), Wan Fang Database, Chinese Biomedical Literature Database (Sino-Med) and VIP Chinese Science and Technology Periodical Database (VIP). The search strategy will be based on the guidance of the Cochrane handbook.

The following searching terms as abstract terms and MeSH Terms will be used including “Chinese herbal medicine” OR “Chinese medicine” OR “Traditional Chinese medicine” OR “Chinese medicinal” OR “Chinese patent medicine” OR “Chinese medicine preparation” AND (“Colorectal cancer” OR “Colorectal carcinoma” OR “Colorectal tumor” OR “colon cancer” OR “rectal cancer” OR “CRC”) AND “random.”

All relevant publications, including dissertation and conference papers, will be researched to ensure a comprehensive search. Clinical Trials.gov (http://www.clinical.trails.gov) and Chinese Clinical Trial Registry (http://www.chictr.org/cn/) will also be searched to identify ongoing or completed clinical trials. Moreover, any additional relevant studies will also be retrieved from the reference lists of systematic reviews and included studies.

### 2.3 Inclusion criteria

#### 2.3.1 Types of studies

All the randomized controlled trials that investigated the effectiveness and safety of CHM combined with chemotherapy for the treatment of patients with CRC will be included. Quasi-randomized trials and observational studies will be excluded. No language restriction will be imposed.

#### 2.3.2 Types of participants

This study will include colorectal patients undergoing chemotherapy, who are confirmed cytologically or pathologically regardless of their gender, the course and severity of the disease, ethnicity, education and economic status and whether or not they are outpatients or inpatients. It is important to note that these participants must be over 18 years of age. Studies that included participants with other cancers or other diseases will be excluded. Diagnostic criteria will be based on NCCN clinical practice guidelines in Oncology: Rectal Cancer (2020.V6) and colon cancer (2020.V4).^[20,21]^ Besides, we also refer to the guideline for the diagnosis and treatment of CRC issued by the Chinese Society of Clinical Oncology.^[22]^

#### 2.3.3 Types of interventions

The intervention group will be treated with oral administration CHM combined with chemotherapy. The CHM in this study includes decoction, tablet, pill, powder, Chinese herbal compound prescription, single CHM and Chinese patent medicine. For studies using other treatments as the third arm, only the 2 arms using CHM and/or chemotherapy will be included for analysis. Comparators will include a placebo for herbal therapy, conventional chemotherapy, or no additional intervention. Co-interventions are conventional chemotherapy for CRC plus usual care. The conventional therapies will be required to be the same in each group.

#### 2.3.4 Types of outcome

##### 2.3.4.1 Primary outcomes

The primary outcome measures will include the following:

(1) Overall Survival: It is defined as the time from allocation until death from any cause.
(2) The 1-, 3-, and 5-year survival rates: The survival rates are defined as the proportion of survived patients at corresponding time-points.

##### 2.3.4.2 Secondary outcomes

Secondary outcomes will include:

(1) Progression-free survival: It is defined as the time from allocation to disease progression or death.
(2) Objective response rate: It is defined as the proportion of patients with a reduction in tumor burden of a predefined amount.
(3) Side effects associated with chemotherapy: Side effects include nausea, vomiting and neurotoxicity caused by chemotherapy.
(4) Karnofsky performance status: The Karnofsky score was used to assess the performance status.
(5) The adverse reactions rate of CHM.

### 2.4 Exclusion criteria

(1) The unrelated and duplicated documents will be deleted.
(2) Reviews, theoretical discussions, experience summaries, and case reports will not be considered.
(3) Articles lacking raw data will not be included.

### 2.5 Data collection and extraction

**Figure 1:**
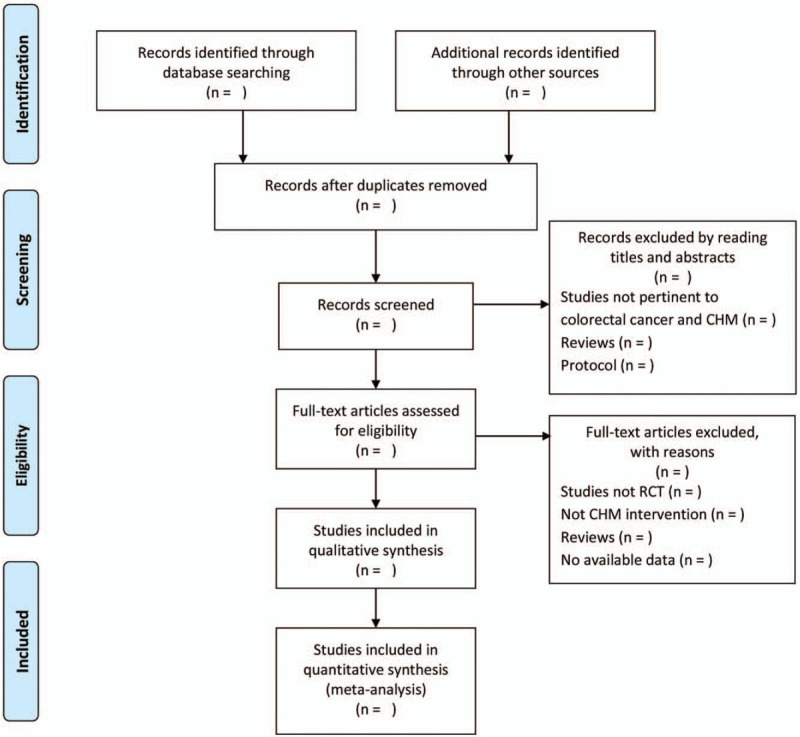
Flow diagram of the study selection process.^[31]^ (Arrows = flow directions or reasons for exclusion of trials, RCT = randomized controlled trial.).

Study screening will be based on the Cochrane Handbook for Systematic Reviews of Interventions.^[23]^ NoteExpress software (version:3.2; Beijing Aegean Software Company, Beijing, China) is used to retrieve the import of screening results,Eliminate the duplicate literature, and exclude irrelevant literature by reading titles and abstracts. We will then read the full text to retain the randomized clinical trials that meeting the inclusion criteria. The selection process is shown in a Preferred Reporting Items for Systematic Review and Meta-Analysis Protocols flow chart (http://www.prisma-statement.org/) (Fig. 1).

Data extraction will refer to the Cochrane Handbook for Systematic Reviews of Intervention (V.5.1.0).^[23]^ Two reviewers (LWY and GJ) will extract the data independently by a self-developed and standardised data extraction form. Any disagreement in the process will be resolved by discussion with another team member (WQL). If insufficient or ambiguous data is reported during data extraction, 2 reviewers will contact the corresponding author of the clinical trial by email to request additional information.

Data extraction contents will include the following details:

(1) General information: Study ID (the author, year of publication), title, publication status, report sources, and fund support;
(2) Methodological information: design type, grouping, random sequence generation, allocation concealment, blinding, number of lost during follow-up, selective reporting and baseline condition;
(3) Participant information: diagnostic criteria, inclusion criteria, exclusion criteria, source of patients, population, sample size, age, gender, course of disease, and tumor stage;
(4) Intervention information: details of the intervention, syndrome differentiation of Traditional Chinese Medicine, dosage form, conventional treatment, duration of treatment, and clinical follow-up;
(5) Outcomes;
(6) Adverse events.

### 2.6 Assessment of risk of bias

The Cochrane risk of bias assessment tool will be used.^[24]^ The risk of bias will be assessed as follows: random sequence generation, allocation concealment, blinding of participants and personnel, blinding of the outcome assessment, incomplete outcome data, selective outcome reporting, follow-up, and other sources of bias. Two reviewers (LWY and GJ) will independently assesse the risk of bias for included studies. “L,” “H” and “U” will be used as a code for the evaluations of the above bias risks. “L” indicates a low risk of bias, “H” indicates a high risk of bias, “U” indicates that the risk of bias is unclear. The trials will be marked as L, H or U in each evaluation range according to its methodological quality. Disagreements will be resolved by discussion and consensus. If necessary, we will contact the authors of the included studies to inquire about some missing information.

### 2.7 Data synthesis and analysis

Review Manager Software (RevMan, Version 5.3 for Windows, The Cochrane Collaboration, Oxford, England) will be applied to analyze and synthesize the outcomes systematically. The quantitative synthesis will be done if clinical heterogeneity is not considered by at least 2 authors in the discussion. The continuous variable will be described by mean difference, *P-*value and 95% confidence interval. Relative risk, *P-*value and 95% confidence interval will be adopted to describe the dichotomous outcomes. *I*^2^ test will be used to judge the statistical heterogeneity of meta-analysis. I^2^ value > 50% will be considered as an indication of substantial heterogeneity. In this case, the data will be analyzed with a random-effect model and the possible causes of heterogeneity will be examined. Otherwise, the fixed-effect model will be adopted. If there is significant statistical heterogeneity, the cause of heterogeneity should be explored. Sensitivity analysis and subgroup analysis will be carried out when necessary.

#### 2.7.1 Addressing missing data

For the missing data, we will contact the corresponding author of the clinical trial by email to get additional information. If sufficient data is not available, an intention-to-treat analysis will be performed where feasible.

#### 2.7.2 Subgroup analysis

If the necessary data are available, Subgroup analysis will be performed according to these characteristics: pathological stage, the severity of included patients, different types of CHM interventions, treatment duration, and outcome measures.

#### 2.7.3 Sensitivity analysis

Sensitivity analysis will be adopted to ensure the robustness of study results. The reliability of the conclusions will be determined by the methodological quality, sample size, and choice of using missing data.

### 2.8 Publication bias

The potential publication bias will be analyzed by the funnel plot or Egger test. The funnel plot will be used if the number of trials included in the meta-analysis is more than 10. If the number of included studies is less than 10, the Egger test will be applied. The analysis software of Egger test will be R 3.6.2 for Windows.

### 2.9 Assessment of quality of evidence

This study will evaluate the evidence of clinical outcomes according to the Grading of Recommendations Assessment, Development, and Evaluation standard. This study will consider factors that may reduce the quality of evidence, such as limitations in study design, the inconsistency of results, indirectness of evidence, inaccuracy and publication bias. Grading of Recommendations Assessment, Development, and Evaluation Pro GDT online software (http://www.guidelinedevelopment.org/) will be used to form the summary of findings table (SoF table).

## 3 Discussion

Chemotherapy is 1 of the most common treatments for CRC. For CRC, 5-Fu and oxaliplatin are the main chemotherapy drugs used by oncologists for the treatment of CRC.^[25]^ Due to the general toxicity of the drugs, chemotherapy results in some side effects. Some cancer patients cannot adhere to the effective chemotherapy cycle, the side effects of chemotherapy affect the treatment effect and the QOL.

However, it is encouraging to see that the promise of traditional Chinese medicine in cancer treatment is increasingly being recognized. CHM is 1 of the most common complementary therapies for chemotherapy, surgery and radiotherapy around the world.^[26]^ CHM as an adjunctive therapy has shown obvious advantages in improving the clinical symptoms and the therapeutic efficacy of chemotherapy, as well as in alleviating the side effects of chemotherapy.^[27–29]^ A systematic review published as early as 2012 showed that compared with chemotherapy alone, CHM, as an adjuvant therapy of chemotherapy, has significant efficacy in prolonging survival time, enhancing tumor response, improving QOL, regulating immune function and alleviating acute adverse reactions.^[30]^ In recent years, it is common to use CHM combined with chemotherapy to treat CRC in China, but there are still few published systematic reviews in English, which has hindered the worldwide popularity of CHM. The purpose of this study is to assess the efficacy of CHM as an adjunctive therapy to chemotherapy for patients with CRC. We hope this review will stimulate the proper evaluation of CHM.

## 4 Ethics and dissemination

This review does not require ethical approval because the included studies are published data and the data does not involve the patient's privacy.

## Author contributions

Fengming You and Wenyuan Li participated in the study design. Jing Guo and Qiaoling Wang will develop the search strategies, conduct data collection, and analyze independently. Jianyuan Tang and Fengming You will revise it.

Writing-original draft, writing-review and editing: Wenyuan Li and Jing Guo.

Software: Wenyuan Li, Jing Guo and Qiaoling Wang.

Risk of bias assessment: Wenyuan Li, Jing Guo.

Supervision, revision article: Fengming You.

All authors have approved the final manuscript.

**Conceptualization:** Wenyuan Li, Fengming You.

**Data curation:** Jing Guo, Qiaoling Wang.

**Formal analysis:** Wenyuan Li, Jing Guo, Qiaoling Wang.

**Funding acquisition:** Fengming You.

**Investigation:** Jing Guo.

**Methodology:** Jing Guo, Qiaoling Wang.

**Project administration:** Fengming You.

**Software:** Wenyuan Li, Jing Guo, Qiaoling Wang.

**Supervision:** Fengming You.

**Validation:** Jianyuan Tang, Fengming You.

**Writing – original draft:** Wenyuan Li, Jing Guo.

**Writing – review & editing:** Wenyuan Li, Jing Guo, Jianyuan Tang, Fengming You.
